# Supplementary material for: Chromothripsis during telomere crisis is independent of NHEJ, and consistent with a replicative origin
Source: Genome Res. 2019 May;29(5):737–49. doi: 10.1101/gr.240705.118 (PMC6499312; doi:10.1101/gr.240705.118)
Supplement: Supplemental Material [file supp_gr.240705.118_Supplemental_file_1.zip › contigs/annotated_contigs/DB81/contig.2.DB81_length_633_mean_cov_12.5718799368.docx]

**DB81_length_633_mean_cov_12.5718799368**

```
AATGGAAACGTTCCTTGCTAATTCAACCGTGGAAAGAAAACTTGGCAATTGAGTTTTGGAAAGCAGAGGCTCTTTTATCTACCTGATCA
 >chr1:103660817-103661095 + E=1e-152
ATATAGTATCATCAGTTGACAGATTTATTCCCACATGGTTATTAACTTACTCAAGCAAATGAAAATTTCATGAATATCTAGCAGTCATG

GCAAGATGTTAACAGAGTTTGACAAAGAGATTTAAAATGTATTATGTACAAGTTTCTTATTTCTTATTACAAACTTTTTTTGCTTTACT

GACATGAAC|TG|GCAGATACCTTAAAAAGTAGAAAATAGGAATACTTCTCTTCATTGCCAAAATACATTACTGAATTCTTTTAGTGAT
          >chr1:103659218-103659575 + E=4e-203
GATAGAGTTGTAGGTAGTGCTATAGAAAGTAGAATACGATTGTTATTTTTAATTGCAATAACTCTATAGAACTGTAGAGATTGCTATAA

AATTTACTTTTGTTCTTTAAGCTTTTAGGAAGTACTGAGCAATGAATGTTAGATATCATGAAATAGTGTTGATTTCATAGGCATTTAAA

TATGTACTCTCCTGTATACCTTAGTATTTCACATTTACCAACCTATAATTTTTGGCCAAAGTATACATATTTTCAAATTACAGCTTATA

AAATAACACTTC
```
